# Jasmonic Acid at the Crossroads of Plant Immunity and *Pseudomonas syringae* Virulence

**DOI:** 10.3390/ijms21207482

**Published:** 2020-10-11

**Authors:** Aarti Gupta, Mamta Bhardwaj, Lam-Son Phan Tran

**Affiliations:** 1 Department of Life Sciences, POSTECH Biotech Center, Pohang University of Science and Technology, Pohang 37673, Korea; aarti2785@postech.ac.kr; 2 Department of Botany, Hindu Girls College, Maharshi Dayanand University, Sonipat 131001, India; mamtakaushik2006@gmail.com; 3 Institute of Research and Development, Duy Tan University, 03 Quang Trung, Da Nang 550000, Vietnam; 4 Stress Adaptation Research Unit, RIKEN Center for Sustainable Resource Science, 1-7-19 22, Suehiro-cho, Tsurumi, Yokohama 230-0045, Japan

**Keywords:** effectors, jasmonates, *Pseudomonas syringae*, salicylic acid, stomata

## Abstract

Sensing of pathogen infection by plants elicits early signals that are transduced to affect defense mechanisms, such as effective blockage of pathogen entry by regulation of stomatal closure, cuticle, or callose deposition, change in water potential, and resource acquisition among many others. Pathogens, on the other hand, interfere with plant physiology and protein functioning to counteract plant defense responses. In plants, hormonal homeostasis and signaling are tightly regulated; thus, the phytohormones are qualified as a major group of signaling molecules controlling the most widely tinkered regulatory networks of defense and counter-defense strategies. Notably, the phytohormone jasmonic acid mediates plant defense responses to a wide array of pathogens. In this review, we present the synopsis on the jasmonic acid metabolism and signaling, and the regulatory roles of this hormone in plant defense against the hemibiotrophic bacterial pathogen *Pseudomonas syringae.* We also elaborate on how this pathogen releases virulence factors and effectors to gain control over plant jasmonic acid signaling to effectively cause disease. The findings discussed in this review may lead to ideas for the development of crop cultivars with enhanced disease resistance by genetic manipulation.

## 1. Introduction

An incidence of virulent pathogen infection in a plant, which is incapable of surmounting effective immune responses, leads to the appearance of plant disease. A resistant plant, on the other hand, is well equipped with a versatile arsenal and employs different signaling molecules to recognize and thwart away pathogens. Jasmonic acid (JA) and its derived molecules (mainly, methyl jasmonate, MeJA, and isoleucine-conjugated jasmonate, JA-Ile) are commonly designated as jasmonates (JAs), a subgroup of oxylipins derived from 12-oxo-phytodienoic acid (OPDA) [1]. JAs have been associated with plant defense against herbivory and necrotrophic pathogen attacks [2]. On the other hand, JAs suppress plant resistance responses against biotrophic and hemibiotrophic pathogens, while salicylic acid (SA) is known to promote effective disease resistance responses to these pathogens [3,4]. This antagonism between SA and JAs has been further supported by studies in *Arabidopsis thaliana* where the SA-mediated signaling is noted to suppress the JA signaling, thereby protecting plants against biotrophic or hemibiotrophic pathogen infections [2,3,5,6]. This antagonism is very well exploited by biotrophic and hemibiotrophic pathogens to promote JA synthesis and signaling, thereby weakening the SA-dependent plant defense to infect the host plants [6,7,8,9]. 

However, a few emerging reports have associated JAs with positive regulation of plant immunity during hemibiotrophic pathogen infection [10]. Besides, hemibiotrophic pathogen infection increases endogenous JA concentrations in *Arabidopsis* [11]. From the published reports, it is now apparent that JA action can be dependent or independent of any SA functioning, while modulating plant immunity to hemibiotrophic pathogen infections. Thus, the regulatory roles of JAs in plant resistance responses under biotrophic and hemibiotrophic pathogen infections appear to be very enigmatic. In this review, the biological functions of JAs and associated signaling are discussed with respect to the plant immunity against the infections by hemibiotrophic pathogens, and how these pathogens can modulate JA signaling. *Pseudomonas syringae* is a major hemibiotrophic bacterial pathogen, which wreaks havoc in a multitude of host plants, such as *Arabidopsis*, tomato (*Solanum lycopersicum*), and tobacco (*Nicotiana tabacum*). This review encompasses the regulatory roles of JAs in plant immunity, by mostly reviewing the latest literature on the infections of *P. syringae* in various plant species.

## 2. JA Metabolism

**Figure 1:**
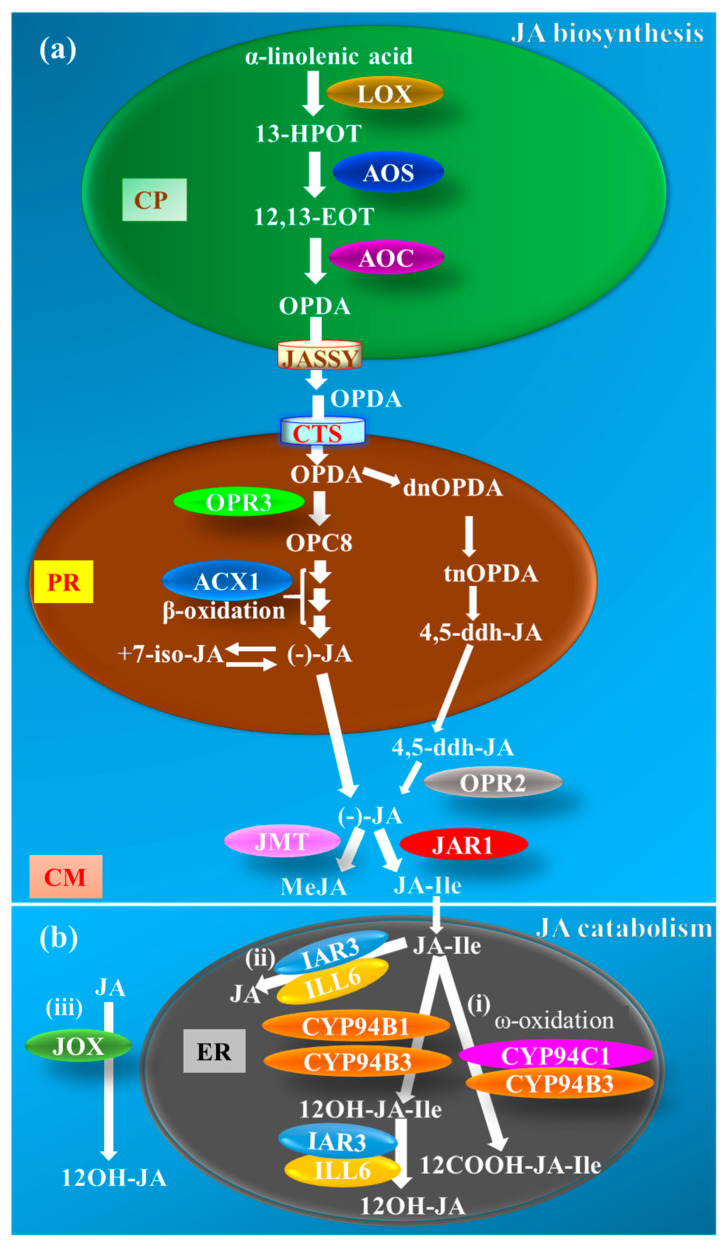
Metabolism of jasmonates (JAs). (**a**) Jasmonic acid (JA) is synthesized from α-linolenic acid by the various enzymes present in chloroplasts (CPs) and peroxisomes (PRs) [12]. LOX, AOS, and AOC are the key enzymes of JA biosynthesis present in CPs. These enzymes convert α-linolenic acid into OPDA through a series of reactions. OPDA is then exported from the CPs by the JASSY transmembrane protein present on the inner membrane of the CPs [13]. OPDA enters into PRs through an ABC transporter membrane protein known as COMATOSE (CTS) [14]. OPDA is directly converted to JA in the PRs with the help of OPR3 and ACX1 enzymes through β-oxidation, and then transported into the cytoplasm (CM) [12]. In the absence of a functional OPR3 enzyme, OPDA is converted to 4,5-ddh-JA, which is then transported into the CM [18,19]. The 4,5-ddh-JA is now converted to JA through the OPR2 enzyme in the CM. In the CM, JAR1 and JMT act on the JA and convert it into JA-Ile [16] and MeJA [15], respectively. (**b**) JA catabolism is regulated by CYP94B1, CYP94B3, and CYP94C1 present in the endoplasmic reticulum (ER) and amidohydrolases IAR3 and ILL6 [22]. (**i**) CYP94B1 and CYP94B3 degrade JA-Ile to the weak analog 12OH-JA-Ile, while CYP94B3 and CYP94C1 catalyze JA-Ile to the inactive form 12COOH-JA-Ile [21,22,23,24,25,26] (**i**). JA-Ile is also converted to JA with the help of IAR3 and ILL6 enzymes [28] (**ii**). These enzymes also work downstream to the CYP94s and breakdown 12OH-JA-Ile generated from step (**i**) to 12OH-JA. In an alternative catabolic route, JOX hydroxylates JA to 12OH-JA [27] (**iii**). ACXI, ACYL-CoA-OXIDASE 1; AOC, ALLENE OXIDE CYCLASE; AOS, ALLENE OXIDE SYNTHASE; 12COOH-JA-Ile, 12-carboxy-jasmonic acid isoleucine; CYP94, CYTOCHROME P450 MONOOXYGENASE CYP94 FAMILY; 4,5-ddh-JA, 4,5-didehydro-jasmonic acid; dnOPDA; dinor-oxo-phytodienoic acid; 12,13-EOT,12,13-epoxyoctadecatrienoic acid; 13-HPOT, 13-hydroperoxyoctadecatrienoic acid; IAR3, INDOLE ACETIC ACID (IAA)-ALANINE RESISTANT 3; ILL6, IAA-LEUCINE RESISTANT (ILR)-LIKE GENE 6; JA-Ile, isoleucine conjugated jasmonate; JAO, JASMONIC ACID OXIDASE; JAR1, JASMONATE RESISTANT 1; JASSY, a membrane channel that is located in the outer envelope of chloroplasts; JMT, S-ADENOSYL-L-METHIONINE: JASMONIC ACID CARBOXYL METHYLTRANSFERASE; LOX, 13-LIPOXYGENASE; MeJA, methyl jasmonate; 12OH-JA, 12-hydroxy-jasmonic acid; 12OH-JA-Ile, 12-hydroxy-jasmonic acid isoleucine; OPC8, 3-oxo-2(2′[Z]-pentenyl)-cyclopentane-1-octanoic acid; OPDA, 12-oxo-phytodienoic acid; OPR, OPDA REDUCTASE; tnOPDA, tetranor-oxo-phytodienoic acid.

The JA biosynthesis pathway in different plant species was first construed by Vick and Zimmerman [12], which is catalyzed in three different locations, starting from chloroplasts to peroxisomes and culminating in the cytoplasm (Figure 1a). Briefly, JA biosynthesis initiates with the 13-LIPOXYGENASE (LOX)-mediated oxygenation of α-linolenic acid (18:3) in the chloroplast membrane. Subsequent reactions catalyzed by ALLENE OXIDE SYNTHASE (AOS, a CYTOCHROME P450 CYP74) and ALLENE OXIDE CYCLASE (AOC) generate cyclopentenone OPDA (18 carbons) and dinor-oxo-phytodienoic acid (dnOPDA, a 16-carbon homolog of OPDA) (Figure 1a). The OPDA is then exported from chloroplasts by JASSY (a membrane channel that is located in the outer envelope of chloroplasts) [13] and imported into peroxisomes by COMATOSE (CTS), an ABC transporter located in the peroxisome membrane [14]. Subsequently, OPDA REDUCTASE 3 (OPR3) converts OPDA to 3-oxo-2(2′[Z]-pentenyl)-cyclopentane-1-octanoic acid (OPC8), which in turn, undergoes three consecutive reactions of β-oxidation catalyzed by ACYL-CoA-OXIDASE 1 (ACX1) to produce JA (Figure 1a). The nascent JA produced in peroxisomes is eventually translocated to the cytoplasm for various metabolic alterations, including hydroxylation, decarboxylation, glycosylation, methylation, and amino acid conjugation [15,16]. JA is methylated by S-ADENOSYL-L-METHIONINE:JASMONIC ACID CARBOXYL METHYLTRANSFERASE (JMT) to form MeJA [15] (Figure 1a). Besides, JA also undergoes amino acid conjugation by the action of JASMONATE RESISTANT 1 (JAR1), a jasmonate-amido synthetase, to yield JA-Ile [(+)-7-*iso*-JA–L-Ile] [16] (Figure 1a). MeJA and JA-Ile are the most common biologically active JA forms reported in plants [17]. In *Arabidopsis opr3*, mutants that lack a functional OPR3-dependent JA biosynthetic pathway, the OPDA is demonstrated to adopt an alternate route and undergoes β-oxidation reactions to generate 4,5-didehydro-jasmonic acid (4,5-ddh-JA, an OPDA derivative), which is released into the cytoplasm where it is reduced by OPR2 in an OPR3-independent pathway to synthesize JA [18,19] (Figure 1a).

The JA catabolism has been well-demonstrated in higher plants for its role in maintaining the JA homeostasis during a switch from inducible to non-inducible conditions. Three distinct JA-catabolic pathways are well demonstrated in *Arabidopsis* (Figure 1b). In the first pathway, JA catabolism is majorly attributed to the hydroxylation of JA-Ile by the CYTOCHROME P450 MONOOXYGENASE CYP94 family members in an ω-oxidation reaction [20,21,22,23,24,25]. The CYP94B3 (a jasmonoyl-isoleucine-12-hydroxylase) and its less predominant homolog CYP94B1 catalyze mono-oxygenation of JA-Ile to a weak analog, 12-hydroxy-jasmonic acid isoleucine (12OH-JA-Ile), whereas CYP94C1 along with CYP94B3 catalyze the double oxygenation of JA-Ile to yield an inactive form, 12-carboxy-jasmonic acid isoleucine (12COOH-JA-Ile) [21,22,23,24,25]. Both CYP94B3 and CYP94C1 reside on endoplasmic reticulum (ER), and hence, associate JA inactivation with the ER site [22] (Figure 1b, (i)). The second pathway involves the deconjugation of the JA–amino acid conjugates by amidohydrolases encoded by INDOLE ACETIC ACID (IAA)-ALANINE RESISTANT 3 (IAR3)- and IAA-LEUCINE RESISTANT (ILR)-LIKE GENE 6 (ILL6), which cleave JA-Ile to free JA and disrupt JA-Ile-dependent signaling [20,21,22,23,24,25,26] (Figure 1b, (ii)). The third alternative route for maintaining JA homeostasis is catalyzed by JASMONATE-INDUCED OXYGENASE (JOX)-mediated direct hydroxylation of JA to the inactive 12-hydroxy-jasmonic acid (12OH-JA) [27] (Figure 1b, (iii)). However, the subcellular location of the enzyme and its action is not known yet. Besides, the amidohydrolases IAR3 and ILL6 also breakdown 12OH-JA-Ile conjugates downstream of CYP94Bs in the first pathway to yield the inactive 12OH-JA [28] (Figure 1b, (i)). Evidence for subcellular location of IAR3 and ILL6 is still lacking; however, based on the fact that these two proteins act downstream of the ER-localized CYP94s and the prediction results obtained using the “Subcellular Localisation Database for Arabidopsis Proteins” database (https://suba.live), we tentatively placed this branch in the ER.

## 3. JA Signaling

CORONATINE INSENSITIVE 1 (COI1) receptor and JASMONATE ZINC-FINGER EXPRESSED IN INFLORESCENCE MERISTEM (ZIM)-DOMAIN (JAZ) proteins are the integral components of the JA signaling [29,30]. COI1 is a nuclear F-box component of an S PHASE KINASE-ASSOCIATED PROTEIN 1 (SKP1)-CULLIN-F-BOX (SCF)-type E3 ubiquitin ligase complex, and JAZs are negative regulators of JA-induced genes and are tagged by the SCF^COI1^ complex for the 26S proteasome-mediated degradation [29,30]. 

**Figure 2:**
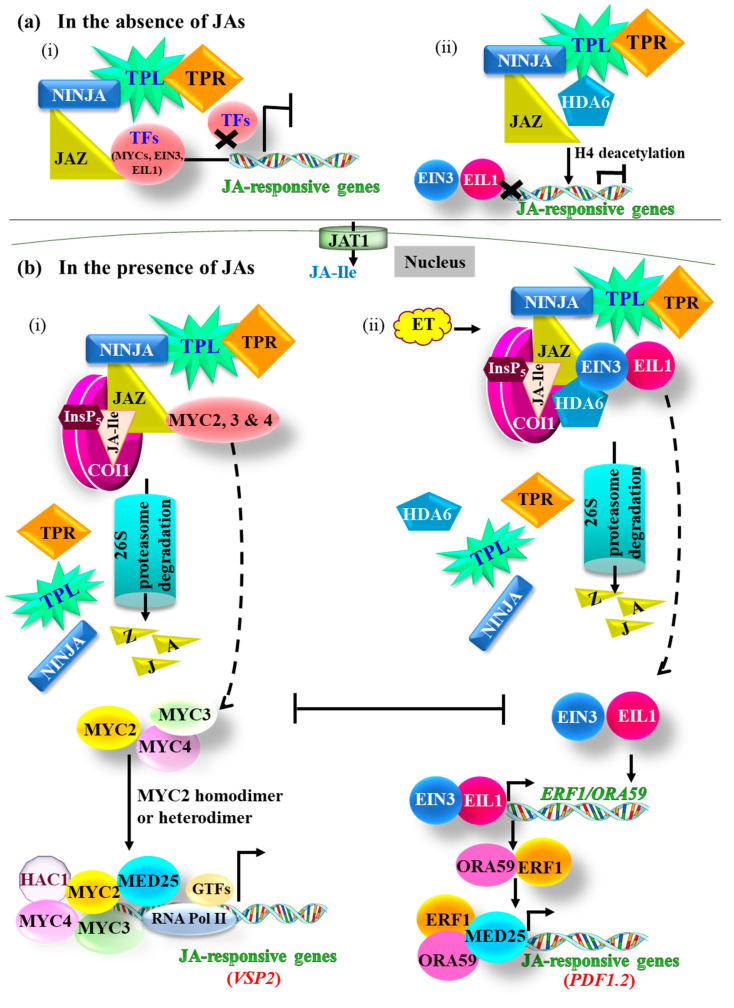
Overview of the jasmonic acid (JA) signaling pathway. (**a**) In the non-inductive phase of JA signaling or in the absence of jasmonates (JAs), JAZ proteins form a complex with the co-repressor TPL and TPR proteins [31]. The association between JAZs and TPL–TPR co-repressors is mediated by the NINJA adaptor protein [32]. The JAZ^NINJA^–TPL–TPR repressor complex then binds to the transcription factors (TFs, such as MYCs, EIN3, and EIL1) and prevents the induction of JA signaling (**i**). JAZ proteins also recruit the transcriptional co-repressor HDA6 protein, which deacetylates histones (especially H4) and inhibits EIN3 and EIL1 to bind to the target gene promoters, leading to the subsequent repression of JA-responsive gene transcription [34,35] (**ii**). (**b**) When a sufficient level of JA-Ile is present in the cells (induction phase), JA-Ile enters into the nucleus through JAT1 membrane transporter [36] and binds to COI1 and JAZ^NINJA^–TPL–TPR repressor complex, leading to the COI1-mediated polyubiquitination of JAZ. The polyubiquitination tags JAZ proteins for 26S proteasome-mediated breakdown, thereby releasing the TFs and JA-signaling repressors from the complex. Inositol pentakisphosphate (InsP_5_), a co-receptor for JA-Ile, stabilizes the association of COI1–JAZ complex [37]. Depending upon the nature of infecting pathogens, the JA signaling operates through either (**i**) MYC or (**ii**) ERF TFs. The regulation of JA signaling in the MYC2 branch involves the association of HAC1 with MYC2 and MED25 proteins and binding of various MYC TFs [38]. This activator complex then induces the transcription of the downstream JA-responsive gene (e.g., *VSP2*) (**i**). The regulation of JA signaling through the ERF branch is mediated by ethylene (ET), EIN3, and EIL1. EIN3 and EIL1 TFs induce the transcription of *ERF1* and *ORA59*. ERF1 and ORA59 TFs, in turn, recruit MED25 to the GCC-box motif in the target gene promoters, leading to the activation of JA-responsive genes (e.g., *PDF1.2*) [39] (**ii**). Double-headed bars show mutual repression, while dotted arrows indicate the release of TFs from the repressor complex. L-arrows and L-bars indicate induction and repression of gene transcription, respectively. Crosses indicate the inability of proteins to bind to DNA sequences. COI1, CORONATINE INSENSITIVE 1 receptor; EIL1, ETHYLENE-INSENSITIVE 3-LIKE 1; EIN3, ETHYLENE-INSENSITIVE 3; ERF, ETHYLENE RESPONSE FACTOR; GTFs, general transcription factors; HAC1, HISTONE ACETYLTRANSFERASE OF THE CBP FAMILY 1; HDA, HISTONE DEACETYLASE; InsP_5_, inositol pentakisphosphate; JA-Ile, isoleucine conjugated jasmonate; JAT1, JASMONATE TRANSPORTER 1; JAZ, JASMONATE ZINC-FINGER EXPRESSED IN INFLORESCENCE MERISTEM (ZIM)-DOMAIN PROTEIN; MED25, a subunit of the MEDIATOR transcriptional co-activator complex; MYC, MYELOCYTOMATOSIS; NINJA, NOVEL INTERACTOR OF JAZ; ORA59, OCTADECANOID-RESPONSIVE ARABIDOPSIS AP2 (APETALA2)/ERF59; *PDF1.2*, *PLANT DEFENSIN 1.2*; RNA Pol II, RNA POLYMERASE II; TPL, TOPLESS; TPR, TPL-RELATED proteins; *VSP2*, *VEGETATIVE STORAGE PROTEIN 2*.

In the absence or scarcity of endogenous JAs, JAZ proteins form a complex with the co-repressor TOPLESS (TPL), TPL-RELATED (TPR) proteins and HISTONE DEACETYLASE (HDA) (Figure 2a). This consolidated repressor complex then binds to and represses the actions of positive regulators of the JA signaling, such as the MYELOCYTOMATOSIS (MYC)-type transcription factors (TFs) that consist of both basic helix-loop-helix (bHLH) and leucine zipper motifs [31]. The association of JAZs with TPL and TPR proteins is mediated by an adaptor protein called NOVEL INTERACTOR OF JAZ (NINJA) [32]. This JAZ^NINJA^-TPL-TPR repressor complex tightly holds the bound TFs and inhibits them from activating the transcription of downstream JA-responsive genes [1,31,32,33] (Figure 2a).

However, upon elicitation, once the JA-Ile accumulates in the cytoplasm, it is imported into the nucleus by JASMONATE TRANSPORTER 1 (JAT1, an ABC transporter) located in the nuclear envelope [36]. The imported JA-Ile, in turn, binds to the COI1 receptor, causing a conformational change in the COI1 receptor (Figure 2b). The COI1–JA-Ile complex then enrolls JAZ proteins where the COI1 instigates the 26S proteasome-mediated degradation of the JAZ repressors [7,30] (Figure 2b). The inositol pentakisphosphate (InsP_5_) molecule functions as a co-receptor for JA-Ile and stabilizes the association of COI1–JAZ complex [9,37] (Figure 2b). As a result of JA-triggered degradation of JAZs and disintegration of the repressor complex, the repression on bound TFs is released, allowing them to induce the transcription of downstream JA-responsive genes [9,29,30,32,33]. Depending upon the nature of the elicitor, the JAZ proteins can bind to different classes of TFs. As a result, JA signaling bifurcates into the MYC and the ETHYLENE RESPONSE FACTOR (ERF) branches [40,41,42,43] (Figure 2b). These two branches are primarily governed by the MYC2 (Figure 2b, (i)), and the ETHYLENE-INSENSITIVE 3 (EIN3) and ETHYLENE-INSENSITIVE 3-LIKE 1 (EIL1) (Figure 2b, (ii)) TFs, respectively, and are mutually antagonistic [39]. MYC2 interacts with EIN3 and EIL1 proteins and inhibits their transactivation activities in inducing the downstream target genes, which thereby represses plant responses primarily governed by the ethylene (ET) [43]. Conversely, EIN3 and EIL1 also interact with and inhibit the transcriptional activity of MYC2, thereby inhibiting MYC2-target gene expression and attenuating JA-governed plant responses [43].

### 3.1. The MYC Branch

The MYC branch specifically regulates the wounding- and insect-induced JA responses and is governed by the MYC2 TF. In addition, other members of the MYC family, specifically MYC3 and MYC4, act in conjunction with MYC2 to activate the transcription of JA-responsive marker genes such as *VEGETATIVE STORAGE PROTEIN 2* (*VSP2*) [40,44] (Figure 2b, (i)). In the presence of stress-induced JA-Ile levels, the COI1-dependent degradation of the JAZ repressor complex relieves its repression activity on MYC TFs (i.e., MYC2, 3, and 4). In turn, the liberated MYC2 binds the interacting domain of the MED25 subunit of the MEDIATOR transcriptional co-activator complex [38,45,46]. This MYC2–MED25 complex then recruits the HISTONE ACETYLTRANSFERASE OF THE CBP FAMILY 1 (HAC1), along with an RNA POLYMERASE II, and other required general TFs (GTFs) to the MYC-bound promoters to initiate JA-responsive transcriptional reprogramming [38,45,46]. MED25 physically and functionally interacts with HAC1, which acetylates lysine 9 (K9) in histone H3, specifically at the MYC2-target promoters, thereby favoring gene activation [46] (Figure 2b, (i)). In contrast, under non-induced conditions, when the endogenous JA-Ile levels are below a threshold, the Jas motif of the JAZ proteins conforms to an extended α-helix structure that binds to the N-terminal JAZ-interacting domain (JID) of MYCs [47] (Figure 2a, (i)). The conformational change in the α-helix of the JAZ domain prevents the binding of MYC2 to the MED25 [47], thereby repressing the JA output. The MED25 also interacts physically with COI1, and thus, the MED25–COI1 complex remains stable under the non-inducible conditions [46].

### 3.2. The ERF Branch

The ERF branch is activated specifically upon necrotrophic pathogen attack and is characterized by the transcriptional activation of the gene encoding a PLANT DEFENSIN 1.2 (PDF1.2) [39,48]. This branch is coordinated by the synergism between ET- and JA-signaling pathways where JA accumulation and perception by COI lead to the breakdown of JAZ proteins and subsequent release of JAZ-bound positive regulators EIN3 and EIL1, and ET-induced signaling stabilizes these two TFs [34]. Under non-induced JA levels, JAZs directly interact with EIN3 and EIL1 and repress their functioning, or JAZ proteins recruit the transcriptional co-repressor HDA6 [34,35] (Figure 2a, (ii)). The HDA6-mediated deacetylation of histones (especially H4) obstructs EIN3 and EIL1 to bind to the target gene promoters, thereby inhibiting the subsequent expression of downstream genes (e.g., *ERF1, OCTADECANOID-RESPONSIVE ARABIDOPSIS AP2* (*APETALA2*)*/ERF-DOMAIN PROTEIN 59* (*ORA59*) and *PDF1.2*) and suppressing JA signaling [34] (Figure 2a, (ii)). Direct interaction of HDA6 with EIN3 and EIL1 has also been demonstrated [34]. In addition to the HDA6, the HDA19-mediated histone modification is also regarded as a key factor in the regulation of JA signaling [49]. In the presence of necrotroph-induced accumulation of JAs, the JAZ proteins are degraded, and the EIN3 and EIL1 TFs are released from the repression by JAZ and HDA6 proteins, allowing them to activate the transcription of *ORA59* and *ERF1* [34,39]. The ORA59 and ERF1 TFs, in turn, recruit MED25 and bind to the GCC-box motif present in the promoter regions of downstream genes via the ERF domain and activate their transcription [50] (Figure 2b, (ii)).

### 3.3. Negative Regulation of JA Signaling

**Figure 3:**
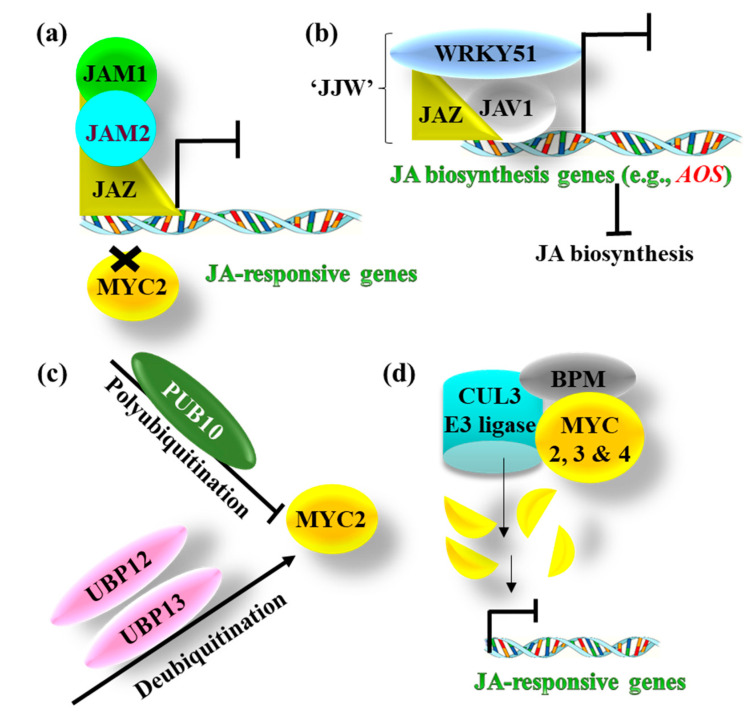
Negative regulation of the jasmonic acid (JA) signaling in *Arabidopsis*. (**a**) JAZs bind to transcription factors (TFs), such as JAM1 and JAM2, and compete with MYC2 for binding to the promoters of JA-responsive genes to repress their transcription [51]. (**b**) JAZ proteins (e.g., JAZ8) also interact with JASMONATE-ASSOCIATED VQ MOTIF GENE 1 (JAV1) and WRKY51 to form a “JJW” co-repressor complex. The JJW complex, in turn, binds to the promoter of JA-biosynthetic genes (e.g., *AOS*) and represses their expression, inhibiting JA biosynthesis [53]. (**c**) MYC2 is polyubiquitinated by PUB10 and is targeted for degradation, while it is stabilized by UBP12- and UBP13-mediated deubiquitination [54,55]. (**d**) The JA-signaling pathway is subjected to feedback regulation by a CUL3^BPM^ E3 ligase system. The BPM proteins interact with MYC2, 3, and 4 and associate these proteins with the CUL3 ^BPM^ E3 ligase complex for ubiquitination and subsequent breakdown. This process leads to the transcription repression of the downstream JA-responsive genes [56]. Bars show repression, and L-bars indicate repression of gene transcription. Cross indicates the inability of proteins to bind to DNA sequences. *AOS*, *ALLENE OXIDE SYNTHASE*; BPM, ((BTB/POZ (BROAD COMPLEX, TRAMTRACK, BRIC-A-BRAC/POX VIRUS, AND ZINC FINGER DOMAIN) and MATH (MEPRIN AND TRAF HOMOLOGY DOMAIN)); CUL3, CULIN3; JAM, JA-ASSOCIATED MYC2-LIKE; JAV1, JASMONATE-ASSOCIATED VQ MOTIF GENE 1; JAZ, JASMONATE ZINC-FINGER EXPRESSED IN INFLORESCENCE MERISTEM (ZIM)-DOMAIN PROTEIN; JJW, JAV1, JAZ, WRKY51; MED25, a subunit of the MEDIATOR transcriptional co-activator complex; MYC, MYELOCYTOMATOSIS; PUB10, PLANT U-BOX PROTEIN 10; UBP, UBIQUITIN-SPECIFIC PROTEASE.

JA signaling is tightly regulated in plant cells and is negatively regulated at multiple stages in the absence of the inducer or during the recovery phase (Figure 3). In addition to binding to MYC2, EIN3, and EIL1 TFs, JAZs have also been shown to bind to other transcriptional regulators belonging to the bHLH class of TFs, such as JA-ASSOCIATED MYC2-LIKE 1 (JAM1) and JAM2, which in turn, bind to the MYC2-target promoters as transcriptional repressors to antagonize MYC2 action and negatively influence JA-dependent responses [51,52] (Figure 3a). It was also reported that JAZ8 interacts with JASMONATE-ASSOCIATED VQ MOTIF GENE 1 (JAV1), which is a suppressor of JA-dependent defense responses, and WRKY51 TF to form a “JJW” co-repressor complex [53]. The JJW complex, in turn, binds to the promoter of the JA-biosynthetic genes (e.g., *AOS* gene) and represses their expression, inhibiting JA biosynthesis [53] (Figure 3b). In addition to the JAZ- or JAM-mediated repression of the MYC2 functioning, the activities of MYC2, MYC3, and MYC4 are also regulated at protein levels. The MYC2 protein is polyubiquitinated by PLANT U-BOX PROTEIN 10 (PUB10), an E3 ligase, and is targeted for degradation, leading to suppression of the JA-responsive *PDF1.2* gene [54]. However, in vitro experiments have shown that the polyubiquitinated MYC2 can be deubiquitinated by UBIQUITIN-SPECIFIC PROTEASE proteins UBP12 and UBP13, which extends the half-life of MYC2 and activates the JA responses [55] (Figure 3c). In a recent study, a negative-feedback regulatory mechanism controlling the MYC2 levels and activity has been suggested [56]. The BROAD COMPLEX, TRAMTRACK, BRIC-A-BRAC/POX VIRUS, AND ZINC FINGER (BTB/POZ) domain and MEPRIN AND TRAF HOMOLOGY (MATH) domain proteins, referred to as the BPM proteins, are the substrate adaptors of CULIN3-based E3 ubiquitin ligases (to form CUL3^BPM^ E3 ligases) and are stabilized by JAs. These BPM proteins interact with MYC2, MYC3, and MYC4 proteins and target the MYCs, particularly MYC2 and MYC3, for CUL3^BPM^ E3 ligases-mediated proteasome breakdown [56] (Figure 3d). In addition, a MYC2-mediated feedback inhibition of JA signaling was demonstrated in tomato in which MYC2 induced the expression of the genes encoding MYC2-TARGETED BHLH (MTB) 1, 2, and 3 proteins [57]. The MTB proteins, in turn, inhibit the formation of MYC2–MED25 complex and compete with MYC2 for binding to the MYC2-target promoters [57]. Hence, the core JA-signaling pathway is subjected to a diverse array of negative-feedback control possibly to tailor spatial and temporal termination of JA output.

## 4. Role of JAs in Plant Defense against *P. syringae* and Some Other Major Hemibiotrophs

The synergism between ET and JA signaling pathways is a requisite for the activation of plant defense against necrotrophic pathogens [39,48,58]. However, such synergism, and JAs themselves, antagonize plant resistance responses to the hemibiotrophic pathogens, specifically *P. syringae* either by meddling with the plant morpho-physiological traits or expression of plant defense-related genes [7,59,60,61,62]. The tomato JA-INSENSITIVE 1 (JIN1), a homolog of *Arabidopsis* COI1, has been implicated as a positive regulator of the hemibiotrophic pathogen *P. syringae* pv. *tomato* DC3000 (*Pto* DC3000)-mediated stomatal reopening, which makes plants more vulnerable to this bacterial strain [7,60,62,63,64]. Notably, the ET- or JA-insensitive *Arabidopsis* mutants display a drastic reduction in the expression of plant defense-related genes (such as *PDF1.2*) and consequently exhibit enhanced resistance against *P. syringae* [58,64,65]. The most predominant plant resistance against hemibiotrophic pathogen depends on the antagonism between JAs and SA, where plants respond to the infecting hemibiotrophic pathogen by activating the SA-dependent defense signaling while suppressing the JA-dependent defense responses [66]. For example, an *Arabidopsis coi1* mutant displays enhanced SA levels and transcript level of the SA-inducible *PATHOGENESIS-RELATED PROTEIN 1* (*PR1*) gene, as well as increased resistance to *Pto* DC3000 infection [67].

### 4.1. JAs Inhibit Plant Immunity

The JAZ repressors are important mediators of early basal and subsequent secondary plant defense responses [64,68]. For example, the *Arabidopsis jaz5/10* double mutant plants display aggravated chlorotic symptoms with increased growth of *P. syringae*, suggesting that both JAZ5 and JAZ10 mutually function to restrict *P. syringae* proliferation [68]. Another study reported that JAZ2 is stably expressed in guard cells and regulates stomatal aperture upon *P. syringae* infection [64]. *Arabidopsis jaz2* mutant plants are partially impaired in a microbe-associated molecular pattern (MAMP), from the crude extract of *Pto* DC3000-induced stomatal closure, and are susceptible to *Pto* DC3000 [64]. Furthermore, it was demonstrated in this study that *Pto* DC3000 produced a phytotoxic virulence factor coronatine (COR), which led to the degradation of JAZ2 and the release of JAZ-repressed MYC2 functioning [64].

In addition, the JA signaling regulates plant response to invading *P. syringae* by inhibiting SA-dependent responses. The suppressive effects of JAs on SA biosynthesis or signaling are mostly mediated by the MYC2. Reportedly, MYC2 represses SA signaling in *Arabidopsis* as evidenced by the fact that *myc2* mutants exhibit increased SA levels, upregulated *PR1* expression, and enhanced resistance to *Pto* DC3000, compared with wild-type plants [67]. Similarly, the deletion of the JA receptor COI1 or MYC2 results in the enhanced levels of endogenous SA and improved resistance to *P. syringae* [62]. MYC2 binds to the promoters of genes encoding NO APICAL MERISTEM (NAM), *Arabidopsis thaliana* ACTIVATING FACTOR 1/2 (ATAF1/2), and CUP-SHAPED COTYLEDON 2 (CUC2) domain NAC TFs, such as ANAC019, ANAC055, and ANAC072 in *Arabidopsis*, and the two homologous NAC TFs JASMONIC ACID 2 (JA2) and JA2-LIKE (JA2L) in tomato, and activate their transcription [61,63,69]. These NAC TFs, in turn, repress the transcription of the SA biosynthesis-related gene *SALICYLIC ACID INDUCTION DEFICIENT 2* (*SID2*, encodes an ISOCHORISMATE SYNTHASE, ICS) but induce the transcription of *BENZOIC ACID*/*SALICYLIC ACID CARBOXYL METHYLTRANSFERASE 1* (*BSMT1*) that encodes an SA-methylating enzyme. This reprogramming of transcriptional regulation leads to reduced SA accumulation, activation of stomatal reopening, and increased *in planta* proliferation of *P. syringae* [61,69]. In addition, EIN3 and EIL1 TFs, which mediate the ERF branch-dependent JA responses (as discussed above in Section 3.2), directly bind to the promoter of the *SID2* gene and repress its transcription to negatively regulate *Arabidopsis* resistance against *Pto* DC3000 [65]. Moreover, the synergism between ET- and JA-signaling pathways counteracts the suppressive effects of SA on JA signaling through ORA59 [70]. Thus, it is clear that both MYC and ERF branches regulate the JA-mediated responses to *Pto* DC3000. In addition to MYC2 and EIN3–EIL1, MITOGEN-ACTIVATED PROTEIN (MAP) KINASE 4 (MPK4) is another node that governs the antagonism between JAs and SA, while positively regulating JA signaling but suppressing the SA signaling [71,72]. The *Arabidopsis mpk4* mutant plants exhibit impaired expression of JA-responsive *PDF1.2* and are insensitive to JAs; however, these mutant plants exhibit increased accumulation of endogenous SA, constitutive expression of SA-dependent *PR1* gene, and consequently are more resistant to *P. syringae* [71,72].

### 4.2. JAs Positively Mediate Plant Immunity

Increasing evidence is also available for the positive role of JA-mediated signaling in regulating plant immunity. For example, the JA-deficient tomato *defenseless-1* (*def*-*1*) mutant plants displayed enhanced susceptibility to the *Pto* DCT6D1 and *Xanthomonas campestris* pv. *versicatoria* DC93-1 [73]. The plant cells exhibit an evolutionarily conserved mechanism to sense and respond to the specific pathogens through the virulence factors (or effectors) released by these pathogens during infection, and convert it to robust defense referred to as the effector-triggered immunity (ETI) [74]. More studies have shown that in addition to the increase in endogenous SA levels, the JA concentration also spikes during ETI [75]. Moreover, a plant infected with *Pto* DC3000 carrying the *avrRpt2* (a type III effector gene encoding a cysteine protease) [66], which causes ETI in the infected sites, did not exhibit the antagonism between JA and SA. Furthermore, during *Pto* DC3000 *avrRpt2*-mediated ETI induction, although the SA accumulates to a very high level, such accumulation does not lead to the inhibition of JA signaling, however [66]. The SA receptors NONEXPRESSER OF PR GENES 1-LIKE PROTEIN 3 (NPR3) and NPR4 positively regulate JA-dependent defense responses, further resulting in activation of the RESISTANT TO *P. syringae* 2 (RPS2)-mediated ETI [10]. In the presence of SA, NPR3 and NPR4 instigate the proteasome-mediated breakdown of JAZ repressors, thereby activating the JA-responsive genes [10].

Not only this, but the relationship between JAs and SA during avirulent *P. syringae* infection also relies on the intracellular ratio of these two hormones, and both JA- and SA-dependent signaling pathways can potentially coexist during a hemibiotrophic infection [76]. For example, the co-treatment of JA and SA at low concentrations leads to the activation of both JA-dependent (e.g., *PDF1.2*) and SA-dependent (e.g., *PR1*) signaling [76]. Similarly, treatment with the metabolic elicitors extracted from *P. fluorescens* N21.4 in *Arabidopsis* activated the expression of genes associated with both JA (e.g., *LOX2* and *PDF1.2*) and SA (e.g., *ICS1*, *PR1,* and *PR2*) pathways as well as quenched the reactive oxygen species (ROS)-mediated oxidative stress and provided protection against *Pto* DC3000 [77]. The MED16 (also known as SENSITIVE TO FREEZING 16, SFR16) subunit of the MEDIATOR complex is another potential candidate that mediates the coexistence of JA- and SA-signaling pathways, where it positively regulates both the SA-dependent plant resistance against the *P. syringae* as well as the JA-dependent resistance responses against the necrotrophic fungal pathogens *Alternaria brassicicola* and *Botrytis cinerea* [78].

JAs not only regulate SA biosynthesis and signaling but also influence the intracellular homeostasis of SA. For example, the MeJA application led to the activation of MYC2-dependent signaling and enhanced the expression of *ENHANCED DISEASE SUSCEPTIBILITY 5* (*EDS5*, also known as *SID1*), encoding a member of the MULTIDRUG AND TOXIN EXTRUSION (MATE) transporter family, which transports SA synthesized in the chloroplasts to the cytoplasm, and reduced expression of *PHYTOALEXIN DEFICIENT 4* (*PAD4*) in a MYC2-dependent manner [79,80,81]. Notably, PAD4 is a positive regulator of EDS5. Thus, JAs inhibit endogenous SA concentration by reducing the expression of *PAD4*. However, in the case of loss-of-function of PAD4 (i.e., *pad4* mutant), JAs positively regulate *EDS5* and stimulate SA accumulation [81]. Therefore, JAs may negatively regulate SA-dependent plant immunity against *P. syringae* in the presence of PAD4 but positively regulate plant defense responses in the absence of PAD4 [81].

In another example, the *Arabidopsis constitutive expression of *vsp1** (*cev1*) mutant, which displays a constitutive expression of the JA-dependent ERF branch-regulated *PDF1*.2 gene, is also more resistant to *P. syringae* pv. *maculicola* ES4326 [82]. In addition, the *B. cinerea* or *A. brassicicola* infection in *Arabidopsis* plants led to the activation of both JA- and ET-signaling pathways [70]. In this case, the induction of the JA- and ET-signaling pathways before the exogenous application of SA rendered the plants to be insensitive to the SA-mediated suppression of JA signaling [70]. In *Arabidopsis,* the functional NPR1 is required for SA-mediated suppression of JA-dependent defenses [70,83]. However, the pharmacological evidence with the application of ET and the ET precursor 1-aminocyclopropane-1-carboxylic acid revealed that ET not only strengthened SA-NPR1-dependent defense responses but also repressed the inhibitory effects of SA on JA-induced *PDF1.2* and *VSP2* expression [83]. It is thus clear that, in addition to the relative levels of the endogenous JAs and SA, the order and chronology of activation of ET-, JA-, and SA-signaling pathways are also crucial factors determining the plant defense responses under the JA and SA crosstalk during pathogen infection.

## 5. *P. syringae* Hijacks JA Signaling to Counter Plant Defense

**Figure 4:**
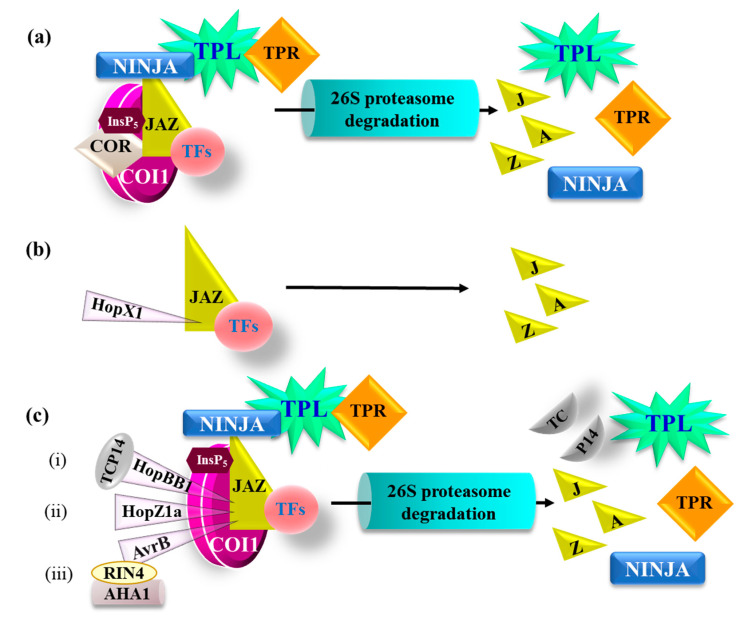
A summary of the *Pseudomonas syringae* factors manipulating jasmonic acid (JA) signaling. Different strains of *P. syringae* secrete virulence factors or effectors to gain control over the plant JA-signaling pathway. (**a**) *P. syringae* pv. *tomato* DC3000 secretes coronatine (COR), which is a mimic of the JA-Ile and perceived through the COI1–JAZ co-receptor complex. This COR–COI1–JAZ complex then directs JAZ proteins, such as JAZ2, JAZ5, and JAZ10, for degradation [69,86]. (**b**) *P. syringae* also releases type III effector HopX1, a cysteine protease that has been reported to interact with the ZIM domain of JAZ family members and degrade them [87]. (**c**) HopBB1 is another effector released by *P. syringae* and interacts with both TCP14 and JAZ3, the repressor components of JA signaling, and glues them together to facilitate their degradation in a COI1-dependent manner [85] (**i**). HopZ1a interacts with the C-terminal Jas domain of JAZ proteins and directs their degradation in a COI1-dependent manner [8] (**ii**). Together with RIN4, the AvrB effector activates the plasma membrane ATPase AHA1. This complex then causes an alteration in membrane potential and, through an unknown mechanism, increases the interaction between COI1 and JAZ, ultimately leading to degradation of the JAZ proteins [88] (**iii**). In all these cases, the degradation of JAZ proteins relieves the repression of transcription factors (TFs) and activation of JA signaling, as described in Figure 2, and leads to enhanced pathogen virulence. AHA1, *Arabidopsis* plasma membrane H+-ATPase; COI1, CORONATINE INSENSITIVE 1 receptor; COR, coronatine; JAZ, JASMONATE ZINC-FINGER EXPRESSED IN INFLORESCENCE MERISTEM (ZIM)-DOMAIN PROTEIN; InsP_5_, Inositol pentakisphosphate; NINJA, NOVEL INTERACTOR OF JAZ; RIN4, RESISTANCE TO *P. syringae* pv. *maculicola* 1 (RPM1)-INTERACTING 4; TCP14, TEOSINTE BRANCHED, CYCLOIDEA AND PROLIFERATING CELL FACTORS 14; TPL, TOPLESS; TPR, TPL-RELATED protein.

While plants are well equipped with defense mechanisms to thwart away pathogens, it has also been demonstrated that pathogens employ a cocktail of virulence factors and effectors to manipulate plant defense responses for their own benefits [8,64,84,85]. One of the ways by which biotrophic and hemibiotrophic pathogens make their way into the plant cells is activating host plant JA biosynthesis and/or signaling pathways (Figure 4), which gives them an edge over plant defense machinery during infection by weakening SA-dependent responses [84]. For example, *Pto* DC3000 secretes COR and hijacks JA signaling. COR is a mimic of the bioactive JA-Ile and perceived through the COI1-JAZ co-receptor complex, it directs JAZ proteins, such as JAZ2, JAZ5, and JAZ10, for degradation (Figure 4a). As a result, the MYC branch is activated, which in turn, induces the expression of *NAC* family genes (such as *ANAC019*, *ANAC055,* and *ANAC072*). These NAC TFs, in turn, inhibit SA accumulation and SA-dependent plant resistance responses to facilitate the bacterial infection in the host plants [67,69,86]. *Pto* DC3000 thus hijacks the COI1–JAZ2–MYC2/3/4–ANAC module that is involved in the regulation of stomatal aperture during the infection process [64]. Besides, COR also impedes stomatal closure by suppressing the guard cell-specific NADPH oxidase-dependent ROS production [68]. As opposed to the COI1-dependent functioning of COR, effector HopX1, which encodes a cysteine protease from *P. syringae* pv. *tabaci* 11528, interacts with the ZIM domain of all the members of the JAZ protein family and triggers their breakdown in a COI1-independent manner to activate the JA signaling, favoring *P. syringae* infection [87] (Figure 4b). Additionally, Yang et al. [85] demonstrated that the *P. syringae* type III effector HopBB1 interacts with both TEOSINTE BRANCHED, CYCLOIDEA, AND PROLIFERATING CELL FACTORS 14 (TCP14), a negative regulator of JA signaling, and JAZ3, a repressor component of JA signaling, and glues them together to facilitate their degradation in a COI1-dependent manner. The breakdown of JAZ3 and TCP14 thus activates the JA-signaling pathway that aids bacterial pathogenesis (Figure 4c, (i)). HopZ1a is another effector molecule produced by *P. syringae* pv. *syringae* A2 (*Psy*) and is a member of a widely distributed YopJ effector family of acetyltransferase. Upon *Pto* DC3000 infection in *Arabidopsis,* HopZ1a interacts with JAZ proteins (e.g., AtJAZ2, AtJAZ5, AtJAZ6, AtJAZ8, and AtJAZ12 in *Arabidopsis*) through the conserved C-terminal Jas domain to acetylate JAZ proteins and direct their degradation in a COI1-dependent manner [8] (Figure 4c, (ii)). The activation of JA-signaling by both HopZ1a and HopX1 led to the suppression of SA-dependent defense responses and increased *P. syringae* virulence. Both HopBB1 or HopZ1a could partially complement the virulence defects of a COR-deficient *P. syringae* mutant [8,85], whereas HopX1 could completely rescue the virulence defects of COR-deficiency in *Pto* DC3000 mutant.

*P. syringae* secretes another effector molecule, AvrB. This effector binds to RESISTANCE TO *P. syringae* pv. *maculicola* 1 (RPM1)-INTERACTING 4 (RIN4) to activate *Arabidopsis* H(+)-ATPase (AHA1), a plasma membrane-localized proton ATPase (also known as OPEN STOMATA 2, OST2), which results in changes in membrane potential. Alteration in plasma membrane potential increases guard cell turgor pressure to induce stomatal opening [88]. The AvrB-mediated activation of AHA1 also increases the binding of COI1 to JAZ proteins through an unknown mechanism, causing the degradation of multiple JAZ proteins [88] (Figure 4c, (iii)). This results in the activation of JA signaling and stomatal opening, leading to increased plant susceptibility to *P. syringae* [88]. In addition to interaction with RIN4, AvrB has also been demonstrated to enhance plant susceptibility to a non-pathogenic strain of *Pseudomonas* by disrupting JA signaling in an *rpm1* mutant background [89]. In addition, AvrB also interacts with MPK4 and recruits HEAT SHOCK PROTEIN 90 (HSP90) through an HSP90 co-chaperone REQUIRED FOR MLA12 RESISTANCE 1 (RAR1) and, thus, targets a RIN4–MPK4–RAR1–HSP90 complex to induce JA signaling [90]. Furthermore, AvrB also interacts with and phosphorylates MPK4 [71], and phosphorylated MPK4 in turn binds to and phosphorylates RIN4. The AvrB-activated JA signaling led to enhanced virulence of the infecting pathogens [90].

With building shreds of evidence, it is now apparent that *P. syringae* injects several virulence factors or effector molecules to hijack host JA-signaling machinery. These effector molecules target different components of the JA-signaling pathway and mostly are redundant in their function, which provide flexibility to the infecting pathogens and makes their infection successful. However, the mechanisms of multiple effectors in hijacking the JA-signaling pathway of the host plant are not clear yet, but the multiple effectors clearly provide the bacteria with an efficient arsenal to breakdown the host defense.

## 6. Conclusions and Future Perspectives

JAs are involved in a myriad of biological processes. A diverse range of components of the JA metabolism and JA-dependent signaling have been fairly elucidated; however, their cue-dependent regulatory functions and the links between established regulators and newly discovered components of the JA signaling still need to be explored. The prominent role of JAs in plant immunity is highlighted by the arsenal of effectors and virulence factors used by pathogens to activate the JA signaling pathway in the host plants. Given that the antagonism between JAs and SA is phylogenetically widespread and ancient, the exploitation of this antagonism by the pathogens to manipulate the host plant defense often works against a wide range of plant species. Nonetheless, studies have elaborated on the importance of relative concentrations of the JAs and SA and other spatio-temporal factors influencing the two signaling pathways in deciding the fate of the invading pathogens that are vulnerable to SA-dependent defense (e.g., biotrophs or hemibiotrophs) or those that are generally vulnerable to JA-dependent defense (e.g., necrotrophic pathogens and insects). The literature on JA signaling suggests extensive redundancy among different JAZ protein-mediated signaling modules. JAZ proteins not only regulate distinct TFs and an array of downstream responses based on the nature of the pathogens, the host plant developmental stages, tissues, or cell types, but they are also a major hub for molecular crosstalk and the integration of JAs with other signaling pathways. In addition, given the small number of examples in which most pathogens evolved multiple effectors to target JAZ repressors, these JAZ proteins are the best candidate targets for manipulation to develop better defensive plants. Hence, detailed knowledge of JA signaling opens up exciting opportunities for translational research to improve crop resistance under a multi-attacker situation.
